# Lessons learnt during 20 years of the Swedish strategic programme against antibiotic resistance

**DOI:** 10.2471/BLT.16.184374

**Published:** 2017-10-03

**Authors:** Sigvard Mölstad, Sonja Löfmark, Karin Carlin, Mats Erntell, Olov Aspevall, Lars Blad, Håkan Hanberger, Katarina Hedin, Jenny Hellman, Christer Norman, Gunilla Skoog, Cecilia Stålsby-Lundborg, Karin Tegmark Wisell, Christina Åhrén, Otto Cars

**Affiliations:** a Department of Clinical Sciences, Lund University, Jan Waldenströmsg 35, 20502, Malmö, Sweden.; b Department of Monitoring and Evaluation, Public Health Agency of Sweden, Solna, Sweden.; c Department of Communicable Disease Control, County of Halland, Halmstad, Sweden.; d Department of Communicable Disease Control, County of Värmland, Karlstad, Sweden.; e Division of Infectious Disease, Department of Clinical and Experimental Medicine, Faculty of Medicine, Linköping, Sweden.; f Department of Clinical Sciences, General Practice/Family Medicine, Lund University, Malmö, Sweden.; g Global Health Systems and Policy: Medicines, Focusing Antibiotics, Department of Public Health, Stockholm, Sweden.; h Department of Microbiology, Public Health Agency of Sweden, Solna, Sweden.; i Department of Infectious Diseases, Institution of Biomedicine, Sahlgrenska Academy, Gothenburg, Sweden.

## Abstract

Increasing use of antibiotics and rising levels of bacterial resistance to antibiotics are a challenge to global health and development. Successful initiatives for containing the problem need to be communicated and disseminated. In Sweden, a rapid spread of resistant pneumococci in the southern part of the country triggered the formation of the Swedish strategic programme against antibiotic resistance, also known as Strama, in 1995. The creation of the programme was an important starting point for long-term coordinated efforts to tackle antibiotic resistance in the country. This paper describes the main strategies of the programme: committed work at the local and national levels; monitoring of antibiotic use for informed decision-making; a national target for antibiotic prescriptions; surveillance of antibiotic resistance for local, national and global action; tracking resistance trends; infection control to limit spread of resistance; and communication to raise awareness for action and behavioural change. A key element for achieving long-term changes has been the bottom-up approach, including working closely with prescribers at the local level. The work described here and the lessons learnt could inform countries implementing their own national action plans against antibiotic resistance.

## Résumé

L'utilisation croissante d'antibiotiques et l'augmentation de la résistance bactérienne aux antibiotiques constituent un défi pour le développement et la santé mondiaux. Il est nécessaire de communiquer et de diffuser les initiatives qui parviennent à contenir ce problème. En Suède, la propagation rapide de pneumocoques résistants dans le sud du pays en 1995 a conduit à la formation du Programme stratégique suédois contre la résistance aux antibiotiques, également connu sous le nom de Strama. La création de ce programme a été un point de départ important pour coordonner des efforts sur le long terme afin de lutter contre la résistance aux antibiotiques dans le pays. Cet article décrit les principales stratégies du programme: engagement aux niveaux local et national; suivi de l'utilisation d'antibiotiques afin de prendre des décisions en connaissance de cause; objectif national de prescription d'antibiotiques; surveillance de la résistance aux antibiotiques pour agir au niveau local, national et mondial; observation des tendances de résistance; lutte contre les infections afin de limiter la progression de la résistance; communication afin d'inciter à l'action et au changement des comportements. L'adoption d'une démarche ascendante a été un élément clé pour favoriser les changements à long terme, notamment la collaboration étroite avec les prescripteurs au niveau local. Le travail qui est décrit ici et les enseignements tirés pourraient aider les pays à mettre en œuvre leur propre plan d'action national contre la résistance aux antibiotiques.

## Resumen

El creciente uso de antibióticos y el aumento de los niveles de resistencia bacteriana a los antibióticos son un desafío para la salud y el desarrollo mundiales. Es necesario comunicar y difundir iniciativas de éxito para contener el problema. En Suecia, una rápida propagación de neumococos resistentes en el sur del país desencadenó la formación del programa estratégico sueco contra la resistencia a los antibióticos, también conocido como Strama, en 1995. La creación del programa fue un importante punto de partida de los esfuerzos coordinados a largo plazo para combatir la resistencia a los antibióticos en el país. En este artículo se describen las principales estrategias del programa: labores dedicadas a nivel local y nacional, supervisión del uso de antibióticos para tomar decisiones fundamentadas, un objetivo nacional para las recetas de antibióticos, vigilancia de la resistencia a los antibióticos para la acción local, nacional y global; seguimiento de las tendencias de resistencia, control de las infecciones para reducir la propagación de la resistencia y comunicación para sensibilizar sobre las medidas y el cambio de comportamiento. Un elemento clave para conseguir cambios a largo plazo ha sido en enfoque ascendente, que incluye trabajar estrechamente con los médicos a nivel local. El trabajo aquí descrito y las lecciones aprendidas podrían ofrecer información a los países que implementan sus propios planes de medidas nacionales contra la resistencia a los antibióticos

## ملخص

يشكل الاستخدام المتزايد للمضادات الحيوية وارتفاع مستويات المقاومة البكتيرية للمضادات الحيوية تحديًا يواجه الصحة والتنمية العالمية. لذلك نحن بحاجة إلى توصيل ونشر مبادرات ناجحة لاحتواء المشكلة، حيث أدى الانتشار السريع في السويد لبكتريا المكورات الرئوية المقاومة في الجزء الجنوبي من البلد إلى تشكيل البرنامج الاستراتيجي السويدي ضد مقاومة المضادات الحيوية في عام 1995، والذي يُعرف كذلك باسم Strama. وقد مثّل إطلاق البرنامج نقطة انطلاق هامة للجهود المنسقة الطويلة الأجل لمواجهة مقاومة المضادات الحيوية في البلاد. ويصف التقرير الاستراتيجيات الأساسية للبرنامج كما يلي: الالتزام بالعمل على المستويات المحلية والوطنية، ومراقبة استخدام المضادات الحيوية لاتخاذ القرارات المستنيرة، واستهداف وطني للوصفات الطبية للمضادات المحلية، ومراقبة مقاومة المضادات الحيوية لاتخاذ إجراءات محلية ووطنية وعالمية، ورصد نزعات المقاومة، ومكافحة العدوى للحد من انتشار المقاومة، والتواصل لزيادة الوعي للعمل والتغير السلوكي. ويتمثل العنصر الرئيسي لتحقيق التغييرات على المدى الطويل في اتباع نهج تصاعدي، والعمل بشكل وثيق مع مقدمي الوصفات الطبية على المستوى المحلي. ويمكن للعمل الموضح في هذه الدراسة والدروس المستفادة أن تساعد البلدان على تنفيذ خططها للعمل الوطني ضد مقاومة المضادات الحيوية.

## 摘要

抗生素用量的不断增加和细菌抗生素耐药性水平的不断增强已经成为威胁全球健康和发展的难题。我们需要交流和传播遏制该问题的成功举措。在瑞典南部迅速蔓延的耐药性肺炎双球菌推动了 1995 年瑞典抗生素耐药性应对战略计划（也称作 Strama）的制定。该计划的制定是长期协调努力解决该国抗生素耐药性问题的重要起点。本文描述了该计划的主要战略：致力于从地方和全国层面开展工作；监控抗生素的使用以制定明智的决策；设定抗生素处方的全国性目标；监督抗生素耐药性的地方、全国和全球措施；追踪耐药性发展趋势；控制感染以限制耐药性的传播；加强宣传以增强行动和行为转变意识。实现长期转变的关键因素是采取自下而上的方式，包括与地方层面的处方开具人员开展密切合作。本文描述的工作内容和经验教训可告知其他实施全国性应对抗生素耐药性行动计划的国家。

## Резюме

Все большее использование антибиотиков и повышение резистентности бактерий к антибиотикам являются проблемой для глобального здоровья и развития. Необходимо поддерживать взаимодействие с успешными инициативами по сдерживанию проблемы и распространять о них информацию. В Швеции быстрое распространение резистентных пневмококков в южной части страны послужило причиной формирования в 1995 году шведской стратегической программы по борьбе с резистентностью к антибиотикам, также известной как Strama. Создание программы стало важной отправной точкой для долгосрочных скоординированных усилий по борьбе с резистентностью к антибиотикам в этой стране. В этом документе описываются основные стратегии программы: целенаправленная работа на местном и национальном уровнях, мониторинг использования антибиотиков с целью принятия обоснованных решений, национальная цель по назначению антибиотиков, наблюдение за резистентностью к антибиотикам для принятия действий на местном, национальном и глобальном уровнях, отслеживание тенденций резистентности, контроль инфекций с целью ограничения распространения резистентности, взаимодействие для повышения осведомленности о действиях и изменение поведенческих схем. Ключевым элементом в достижении долгосрочных изменений был подход «снизу вверх» при тесном сотрудничестве на местном уровне с врачами, назначающими лекарственные препараты. Описанная здесь работа и полученный опыт могли бы помочь странам реализовать свои собственные национальные программы действий против резистентности к антибиотикам.

## Introduction

The high global use of antibiotics, the rapid spread of multidrug-resistant bacteria and the lack of new, effective antibiotics has led to an imminent threat to health systems and global development. The responsibility of national governments for taking action to contain antibiotic resistance was reinforced in the global action plan on antimicrobial resistance adopted at the May 2015 World Health Assembly.^1^ A core strategy for controlling resistance is to coordinate efforts through a national action plan. In Sweden, such a plan was first developed in 2000. It built on the work of the Swedish strategic programme against antibiotic resistance, known as Strama, a nationwide structured and continuously evolving collaboration against antibiotic resistance that has been in place since 1995.^2^

The programme was triggered by a rapid spread of penicillin-resistant pneumococci among children in southern Sweden in the early 1990s.^3^ It started as a voluntary network of government authorities and professional organizations and with the formation of multiprofessional groups in local administrative areas.^4^ From the start, the programme applied a One Health approach to antibiotic resistance, working across sectors and multiple disciplines.^5^ Already in 1986, the use of antibiotics in animal feed for growth promotion was banned in Sweden. Since 2012, an intersectoral coordinating mechanism, presently consisting of 25 agencies and organizations within the public health, animal health, food and the environmental sectors, is in place.

**Fig. 1:**
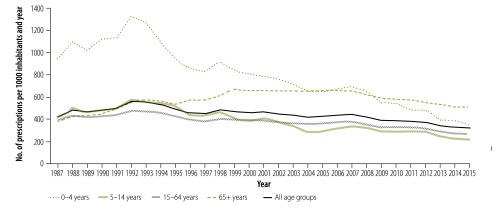
Sales of antibiotics for systemic use in outpatient care, Sweden, 1987−2015

Notes: Includes all antibiotic sales on prescriptions, presented as prescriptions per 1000 inhabitants and year for both sexes by different age groups.

Source: Public Health Agency of Sweden, 2016.^9^

Levels of antibiotic use and resistance in Sweden are now among the lowest among the European Union (EU) countries, both in the human and animal sectors.^6^^–^^8^ Between 1992 and 2016, the number of prescriptions per 1000 inhabitants per year in outpatient care, including primary health care, decreased by 43%, from 560 to 318, while among children aged 0–4 years they decreased by 73%, from 1328 to 349 (Fig. 1).^10^ Adherence to treatment recommendations has been increasing gradually and, notably, sales of antibiotics used for respiratory tract infections have decreased. For several indications there has been a major shift from broad- to narrow-spectrum antibiotics in primary and hospital care, in line with recommendations.^11^^,^^12^ Important factors for change include setting a national target for the number of prescriptions in outpatient care and defining quality indicators based on treatment recommendations, as well as providing local feedback to prescribers.

This paper outlines the stepwise development of the strategic programme against antibiotic resistance in Sweden over a period of 20 years. We describe the structure, key functions and interventions of the initiative across different working areas in human medicine. We hope that the strategies described and the lessons learnt could inform countries starting to implement their own national action plans against antibiotic resistance.

## Commitment at all levels

The government in Sweden is responsible for overall policy on health care, with the responsibility for delivery and financing of health care decentralized to 21 county councils. The financing of the strategic programme against antibiotic resistance has been an evolving process and much of the progress was initially achieved through voluntary work. Funding has gradually increased and become more formalized, now amounting to approximately 2 million euros (€) at the national level, and an estimated € 3–5 million for the work of the local groups.

The local multiprofessional groups function as links between the national and local levels (Box 1). They serve as a mechanism to adapt national initiatives to local conditions. For example by facilitating the implementation of treatment recommendations and by identifying and overcoming barriers to improved antibiotic use and infection control. Typically, they include physicians and other health-care professionals from general practice (primary health care) and departments of infectious diseases, paediatrics, ear nose and throat, clinical microbiology, infection control units and pharmacy. The groups are commonly chaired by the county medical officer for communicable disease control. At the national level the programme has been a forum for all relevant agencies and stakeholders, including health-care professionals, to share experiences and propose initiatives in various areas to counteract antibiotic resistance (Box 2).

Box 1 **Key activities of the Swedish strategic programme against antibiotic resistance at the local level**

- Monitor antibiotic prescribing at the county level and in primary health-care centres, outpatient specialist clinics and hospitals. See Box 3 for systems and tools used.
- Monitor antibiotic resistance within each county in outpatient, including primary health care, and inpatient settings. Surveillance data are generated mainly from routine clinical cultures and to a lesser extent from screening programmes. The scheme is largely dependent on voluntary reporting of local data from microbiological laboratories. See Box 4 for systems and tools used.
- Participate in national point prevalence studies of antibiotic prescribing, including doctor’s diagnoses in outpatient and hospital care, and of health care-associated infections in hospital care.
- Promote the implementation of treatment recommendations via regular, interactive meetings between locally respected members of the strategic programme and each primary health-care centre.
- Communicate new treatment recommendations on common infections and the consequences of antibiotic resistance, both for individuals and for society, to local health-care professionals, the media, decision-makers and the general public.
- Educate staff in primary health-care centres, day-care centres and nursing homes, as well as new parents about infections and the risks and benefits of antibiotics.
- Cooperate with other multiprofessional local groups and the national programme council, to identify common clinical problems, including diagnosis and treatment of infections in daily practice and needed studies or educational activities.
- Develop and implement stewardship programmes in health-care settings. These include a variety of strategies and outcomes, with the general aim of promoting rational use of antimicrobial agents, selection of optimal drugs, dosing, duration of therapy, route of administration and the use of antibiotic susceptibility testing to reduce the use of broad-spectrum antibiotics.^10^

Box 2 **Key activities of the Swedish strategic programme against antibiotic resistance at the national level**

- Coordinate information exchange across the public health, animal health, food and environment sectors. A national mechanism has been in place since 2012.
- Collect, collate and analyse local and national data on antibiotic use and resistance and the health and economic consequences of antibiotic resistance.
- Communicate data to multiprofessional local groups, other health-care professionals, the media, the general public and decision-makers.
- Develop and regularly update evidence-based national recommendations for treatment of common infections in primary health care.
- Initiate national point prevalence surveys of antibiotic prescribing and health care-associated infections in outpatient and inpatient care.
- Analyse collected data to identify gaps between evidence and practice. See Box 3 and Box 4 for systems and tools used.
- Set up and support studies to fill identified knowledge gaps and direct interventions and coordinated actions.
- Support multiprofessional local groups in the implementation of infection treatment recommendations, e.g. by producing locally adapted materials and local educational meetings and events.
- Arrange annual national forums and host a website (http://www.strama.se) as platforms for networking and sharing of best practice among counties.
- Participate in international networks, e.g. European surveillance of antimicrobial consumption network and European antimicrobial resistance surveillance network and international collaborations, including the World Health Organization.

Several lessons were learnt from working jointly with local multiprofessional groups. First, national treatment guidelines must include: diagnostic criteria for each condition; an analysis of the antibiotic risks and benefits both for the patient and for society; and recommendations for when to re-evaluate a patient’s treatment. Second, to ease the implementation of national guidelines in primary health care, they need to be transformed into simple treatment algorithms, e.g. clear advice to health professionals on when and when not to prescribe an antibiotic. Third, it is important to monitor physicians’ prescription of antibiotic, both in primary health care and in hospitals; this will help to stimulate discussions on the ways of improving prescribing practices. Fourth, sustainable funding is required to allocate time for clinical experts to work closely with prescribers, including audit and feedback to achieve increased adherence to guidelines. Fifth, a mandate and financial support from the government is needed, as illustrated by the first five years in which the programme lacked funding and was threatened with closure.

## Monitoring of antibiotic use

### Data access

Statistics on prescriptions and sales of antibiotics from all pharmacies in Sweden are made available monthly, but do not contain any information about diagnoses. National authorities may access data aggregated at the national level, by county or by municipality. Local stakeholders, such as county councils and the programme’s multiprofessional groups, can also access data for each health-care centre and hospital clinic. Available metrics include, prescriptions or defined daily doses per 1000 inhabitants, presented by anatomical therapeutic chemical classification codes. The stepwise development of systems and tools to monitor prescribers’ adherence to antibiotic treatment guidelines are listed in Box 3. In the early 2000, a large number of health-care centres did relatively simple prescription studies, including the given diagnosis.^13^ Since national monitoring systems are lacking in many countries, this way of collecting data on antibiotic use could be a starting point and a driver for change for other countries planning a strategy against antibiotic resistance.

Box 3 **Systems and tools in Sweden to monitor prescribers’ adherence to antibiotic treatment recommendations**

- Point prevalence surveys of infections^13^ and antibiotic use in primary health care (years 2000, 2002 and 2005). Manual registrations were made by general practitioners in 5/21 counties, covering the doctor’s diagnosis, the use of diagnostic tests, symptoms and signs and antibiotic treatment during one week each year.
- System for retrieving data on antibiotic prescribing and diagnosis collected annually (since year 2007) from the primary health-care register of infections in Sweden.^14^ Data are extracted from medical records from 60–90 primary health-care centres, with a listed population of approximately 600 000 inhabitants out of the total population of 10 million.
- Project to develop standardized collection and evaluation of data from electronic medical records in primary health care that is uniform and comparable over time (started at the Public Health Agency of Sweden in the year 2013).^15^
- Annual national point prevalence survey on antibiotic consumption and health care-associated infections in long-term care facilities (started at the Public Health Agency in the year 2014),^16^ with the aim of supporting preventive work.
- Point prevalence surveys of infections and antibiotic use in a large sample of Swedish acute care hospitals by local multiprofessional groups (years 2003, 2004, 2006, 2008 and 2010).^17^
- Point prevalence surveys of health-care associated infections performed by health-care providers and the Swedish Association of Local Authorities and Regions (twice yearly 2008 to 2014 and then annually).
- System for extracting data on the indications for antibiotic treatment, diagnoses and risk factors, and on operations and other patient interventions. Data are extracted from electronic health records at the point of prescription. The anti-infection tool was initiated in 2010 by the Swedish Association of Local Authorities and Regions and was implemented in nearly all Swedish hospitals by 2014. Local multiprofessional groups have an important role in the interpretation and feedback of data to prescribers.

### Evaluation and benchmarking

From an international perspective, antibiotic consumption in Sweden is low. The number of defined daily doses per 1000 inhabitants per day was 12.5 in 2016,^10^ compared with a mean of 22.4 (and the highest level of 36.1) across the 30 EU and European Economic Area countries.^6^ Outpatient care, including primary health care, accounted for the great majority of prescriptions^10^ and was therefore the initial target for the local multiprofessional groups. A decline in Swedish antibiotic consumption started in the mid-1990s and has continued, especially among children aged 0‒4 years (Fig. 1). Specifically, the sales of all antibiotics used for respiratory tract infections decreased. For urinary tract infections, sales of pivmecillinam and nitrofurantoin increased, whereas quinolones and trimethoprim decreased, also in accordance with guidelines on treatment of lower UTI in women, from the Medical Product Agency.^10^^,^^11^ The sales data are consistent with studies of diagnosis-linked data on antibiotic prescribing in primary health care showing a reduction in consultation rates and antibiotic prescribing for respiratory tract infections, especially to children.^9^^,^^18^^–^^20^ Changes in prescribing practices were evident even one year after publication of the new Swedish treatment guidelines.^2^^,^^9^^,^^20^ In 2016, 18.5% (1 824 753) of the Swedish population of 9 851 017 were treated with at least one course of antibiotics.^10^

**Fig. 2:**
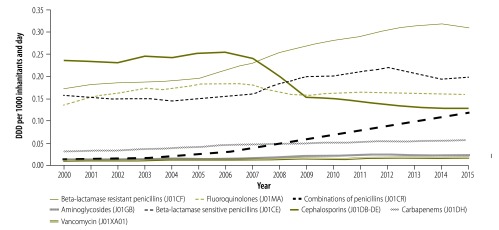
Types of antibiotics commonly used within hospital care, Sweden, 2000−2015

Since 2007, the increasing global spread of extended-spectrum β-lactamase (ESBL-) producing Enterobacteriacae has motivated national and local interventions targeting hospital prescribers.^17^^,^^21^ The result was a substantial reduction between 2007 and 2009 in the use of cephalosporins, and an increase in the use of narrow-spectrum penicillins, which continue to date (Fig. 2). Diagnosis-linked point prevalence studies in Sweden (Box 3) have been instrumental in providing a detailed picture of antibiotic prescribing patterns and patients’ true exposure to antibiotics. Repeated studies between 2003 and 2010 showed that around one-third of all hospitalized patients received antibiotics during each year of the surveys.^17^

DDD: defined daily doses.

Notes: Includes all sales to hospitals, nursing homes and other health-care units. Text in brackets indicate anatomical therapeutic chemical classification codes.

Source: Public Health Agency of Sweden, 2016.^9^

In parallel with the promotion of adherence to the recommendations, national and local registers have been monitored for signs of under-treatment. No increases in serious infection or bacterial complications have been shown that could be attributable to reductions in the prescribing of antibiotics.^22^^,^^23^ In addition, a recent Swedish observational population-based study found that bacterial complications following upper respiratory tract infections were rare, both in patients with no antibiotic treatment and in patients treated with antibiotics. Furthermore, despite the significant decrease in the use of respiratory tract antibiotics in Sweden, bacterial complications following upper respiratory tract infection remained uncommon.^24^

We also learnt several lessons about monitoring. First, diagnosis-linked prescription data are needed for developing targets and regular monitoring of the quality of antibiotic prescribing. Such data, generated by point prevalence studies or retrieved through electronic health records, contribute to the targeting of policy interventions for each clinical condition. Second, the general availability of group A streptococcal tests in combination with clinical criteria are helpful for guiding appropriate antibiotic use in throat infections. However, the lack of rapid tests for identification of other bacterial species and susceptibility is a barrier for targeting empirical therapy, especially in the hospital setting. Third, a national quantitative target for antibiotic use in hospitals is not feasible, because hospitals vary greatly with respect to their size and types of patients attending. Implementation of antibiotic stewardship programmes in hospitals has been slow, and further analyses of the reasons for this are needed. Fourth, when comparing antibiotic use in outpatient care between countries, defined daily doses per 1000 inhabitants and year are influenced by differences in dosage and treatment duration. The number of prescriptions per 1000 inhabitants and year gives more precise information on the number of antibiotic treatments given.

## National target

In 2009, the programme proposed a national long-term quantitative target for antibiotic use in outpatient care. The target was based on epidemiological studies on the frequency of community infections and antibiotic prescriptions from regional studies in primary health care.^18^^,^^19^^,^^25^ Analysis of the data for each diagnosis showed that a large proportion of antibiotic prescriptions was not in accordance with the current recommendations. The national goal was set at no more than 250 prescriptions per 1000 inhabitants per year in outpatient care, taking into consideration a safety margin due to diagnostic uncertainty.

The national goal was included as an indicator of rational prescribing of antibiotics in a Swedish government incentive to improve patient safety (2011–2014). Another indicator included prevention and registration of health care-associated infections. The county councils were eligible to achieve performance-based reimbursement from the state if they met three requirements: (i) support from the county councils to the local multiprofessional groups, including a written mandate and funding; (ii) increased adherence by prescribers to treatment recommendations; and (iii) individual feedback on antibiotic prescribing patterns to general practitioners at more than one half of the health-care centres in the county, and benchmarking of prescribing at all health-care centres. During the initiative, the total sales of antibiotics to outpatients decreased by 15%, from 381 to 325 prescriptions per 1000 inhabitants per year. Number of prescriptions in relation to number of inhabitants dropped in all counties, even though large variations remained between counties.^9^ The target has been used effectively in benchmarking between counties, all of which have shown a decrease.^9^

The lessons learnt from implementing a national target were as follows. First, to make a target credible and accepted it must be: based on epidemiological studies; include data on diagnosis-related prescriptions; comprise a representative patient population; and include evidence-based national guidelines. Second, a target should be regularly re-evaluated using new scientific data, e.g. on changes in incidence of infection. Third, there could be a risk if the target is applied to a too small patient population, e.g. at the health-centre level, as this may include groups that have a greater need for antibiotics. Fourth, a target should be combined with measures to monitor for signs of undertreatment.

## Surveillance of antibiotic resistance

### Data access

Sweden has a long tradition of national surveillance of antibiotic resistance in bacteria of public health concern. This is largely dependent on voluntary reporting of local data from microbiological laboratories.^2^ A national infrastructure with a network of all 26 laboratories using standardized methods and antibiotic susceptibility breakpoints for the past 15 years has allowed this surveillance capacity to be built. The Public Health Agency of Sweden manages the systems and compiles and analyses the data. Surveillance data are generated mainly from routine testing of clinical cultures and to a lesser extent from screening programmes. For outpatients, an annual point prevalence study^26^ has been ongoing since 1994. For patients in hospitals, a point prevalence study over the years 2003‒2010 showed that generally more than two-thirds of the 3000‒5000 antibiotic treatments were preceded by a culture.^17^ This yielded a representative data collection that could be used as basis for treatment guidelines and empirical therapy. Screening is performed for multidrug-resistant bacteria, which are notifiable under the Swedish Communicable Disease Act, primarily in patients who have been hospitalized abroad. Increasing incidence of antibiotic-resistant bacteria and several large hospital outbreaks of resistant bacteria during the last decade have emphasized the need for further development of the automated system for timely response (Box 4). Sweden has participated in the EU’s European antimicrobial resistance surveillance network since 1999.^7^ Sweden has also enrolled in the global antimicrobial surveillance system, launched by the World Health Organization (WHO) in 2016, to support countries in building national surveillance capacity based on quality data and standardized methods.^29^

Box 4 **Systems for monitoring of national data on antibiotic resistance in Sweden**

**National web-based surveillance system for notifiable pathogens**

The system is run in collaboration with the Public Health Agency of Sweden and county medical officers. Microbiological laboratories and responsible physicians report detected cases using a web-based form. The following bacteria and antibiotic resistance combinations are notifiable according to the Swedish Communicable Diseases Act: methicillin-resistant *Staphyloccous aureus*, extended-spectrum β lactamase-producing Enterobacteriaceae, carbapenemase-producing Enterobacteriaceae, vancomycin-resistant enterococci and penicillin non-susceptible Streptococcus pneumonia. As an important complement to the notifications, isolates, excluding extended-spectrum β lactamase-producing Enterobacteriaceae, are sent for epidemiological typing. To ensure an adequate response to the monitoring, results are communicated to laboratories, physicians, hospital management, decision-makers, authorities and concerned organizations as well as the wider public.^10^^,^^27^

**Electronic system for automated daily collection of antibiotic-susceptibility testing results**

The results provide early warnings of defined resistances, including coexisting resistances towards multiple antibiotics, from participating laboratories.^2^^,^^28^ In 2016, data for national reporting clinical isolates from humans were collected through the system and currently 15 of 26 laboratories deliver data. Participation is voluntarily. It was developed by the Public Health Agency of Sweden and representatives of the microbiological laboratories. Relevant data are extracted and sent to the European antimicrobial resistance surveillance network, the European Centre for Disease Prevention and Control system for data on invasive isolates and to the World Health Organization global antimicrobial surveillance system.

**System for national annual point prevalence surveillance to assess resistance patterns**

Data are collected in outpatient settings nationally and locally and include quantitative measures for local quality assurance.^29^ Laboratories compile quantitative data (zone diameters) of at least 100 consecutive clinical isolates from a selection of commonly occurring bacteria and commonly used antibiotics. All laboratories in Sweden have participated voluntarily since the introduction of a national scheme in 1994.

### Tracking resistance trends

To track trends in antibiotic resistance to guide interventions, microbiology sampling in outpatient care is generally performed after treatment failures or prolonged illnesses. A comparatively high number of samples are obtained in primary health care, making it possible to follow resistance patterns and to monitor the development of resistance in relation to national treatment recommendations. With relatively low levels of resistance and only slight increases for some pathogen–antibiotic combinations,^10^ narrow-spectrum antibiotics can be used for most infections in outpatient care in Sweden. The prevalence of EBSL-positive *Escherichia coli* in 26 732 urine samples was 5.6% (1497) in 2016, and methicillin-resistant *Staphylococcus aureus* in 8379 skin and soft tissue infections was around 2% (151). For pneumococci, mostly isolated from nasopharyngeal cultures, a decrease or levelling out of resistance to commonly used antibiotics has been seen since 2010.^10^ Penicillin-resistant pneumococci (minimum inhibitory concentration > 1.0) are scarce, with 67 cases reported in 2016.^10^ According to a nationwide study, levels of community carriage of resistant bacteria in Sweden were comparably low, at 4.9% (101/2134) for fecal carriage of EBSL-producing *E. coli*,^30^^,^^31^ although comprehensive national as well as global data are lacking.^32^

National guidelines for treatment of bacterial infections have been adapted according to the resistance trends. For example, penicillin was kept as the first choice for treatment of community-acquired pneumonia in hospitals. While trimethoprim and quinolones were removed as the first choice for treatment of uncomplicated urinary tract infection, due to high resistance levels in *E. coli* and the negative ecological consequences. The general levels of resistance in Swedish hospitals are increasing only slowly. This could be attributed to the long-term systematic work to maintain appropriate antibiotic prescribing, high use of bacteriological cultures and good compliance with basic hygiene and infection control measures.^2^^,^^10^ In Sweden, as internationally, there is a steady increase of resistance among Gram-negative bacteria. These infections are often acquired by patients during travel to countries with higher incidence of antibiotic-resistant bacteria. This is especially evident for carbapenemase-producing Enterobacteriaceae, even though these strains are still rare in Sweden.^9^^,^^33^ Surveillance data from 2106^10^ showed that, among Gram-positive invasive isolates, methicillin-resistant *Staphylococcus aureus* levels remained stable at 1–2% (11/2163 to 62/2687) over the previous decade. As well as 7% (64/918) of pneumococci had reduced susceptibility to penicillin G; one case of vancomycin-resistant enterococci was found.

Some lessons were also learnt concerning surveillance. First, good quality data on antibiotic resistance are essential to guide empirical therapy. These need to be collected in both hospital and primary health-care settings and by standardized methods. Second, routine clinical cultures have been sufficient as a basis for development of national and local treatment guidelines in Sweden. Third, as cultures are done only on selected cases, the level of antibiotic resistance in the general population may be an overestimate. Screening of outpatients may be important in countries with higher incidence of antibiotic-resistant bacteria than Sweden. Fourth, continuous interaction between local laboratories and multiprofessional groups within the programme is essential to ensure that surveillance is useful for both health-care professionals and decision-makers. Fifth, participation in international networks and surveillance systems is important for monitoring trends and for promoting quality improvements.

## Infection control

Infection control is crucial to prevent infections and limit the spread of resistant bacteria in hospitals and nursing homes. The county medical officers in Sweden have a public authority status and an overall responsibility for prevention and control of communicable diseases within the county. Additionally, in each county, a unit for infection prevention and control is responsible for education of health-care professionals; drafting of clinical routines; assessing levels of health care-associated infections; outbreak control; and collaborations with corresponding clinics at the hospitals. Eight factors correlated with success were identified and may be used by counties, hospitals, clinics and units to draw up action plans to reduce the frequency of health care-associated infections.^34^ These included: (i) awareness that health care-associated infections are unacceptable; (ii) unhesitating compliance with hygiene regulations; (iii) risk assessments enable proactive working methods; (iv) creating favourable physical conditions; (v) consistent message and regular feedback; (vi) cleaning services regarded as vital; (vii) hygiene services and the organization collaborate closely; and (viii) focused management that uses effective channels of communication.^34^

Basic hygiene regulations including dress codes are mandated at the national level and compulsory in health care for all staff. In Swedish national guidelines alcohol hand rub was recommended already in 1973 and is in accordance with WHO guidelines.^35^

Outbreaks of resistant bacteria have occurred in Swedish health-care settings,^36^^–^^38^ including across counties, for example of vancomycin-resistant *Enterococcus faecium*.^39^ Outbreaks in hospitals have been successfully managed by reinforcing existing hygiene routines and the use of single hospital rooms with designated bathrooms. Extensive sampling for screening and case-finding have also been important measurements.

The lessons we learnt on infection control were as follows. First, compliance measurements, e.g. observation of basic hygiene behaviour, including a dress code, were introduced early in Sweden, after educational campaigns. Even though initial financial incentives for compliance have been removed, observations are continuously practised today. Second, evidence is needed that prevention is cost–efficient. In Sweden, an estimated 3 billion Swedish krona are saved annually by decreasing the number of health care-associated infections and hence the hospital beds needed to treat these.^40^ Third, it is increasingly difficult to maintain favourable physical conditions for minimizing transmission of bacteria in health-care settings due to limited financial resources and hospital beds.

## Communication

Awareness-raising and education for both prescribers and the public have been important components in the promotion of rational use of antibiotics (Box 5). Patient’s expectations, as well as doctor’s perceptions of these expectations, can influence antibiotic prescribing.^42^^–^^44^ The Swedish population has a fairly good knowledge and awareness about the use of antibiotics and implications of resistance.^45^^,^^46^ In a European survey, Swedish participants had among the highest overall knowledge of the participating countries, with 98% of the 1035 participants agreeing with the statement “unnecessary use of antibiotics make them become ineffective.”^47^ Half of the 1035 Swedish respondents recalled receiving information on “not taking antibiotics unnecessarily e.g. in the case of cold or flu” in the last 12 months.

Box 5 **Examples of awareness raising and educational activities on antibiotic resistance in Sweden**

- Annual data on consumption and resistance are presented in a coordinated way at the national and local levels in press releases, along with regular reports, studies or important events, to the general public.
- Updated national treatment recommendations are published and available for download from the Swedish medical products agency (http://www.lakemedelsverket.se), in conjunction with articles and opinion pieces produced for magazines for health-care professionals.
- Patient information leaflets on common infections are produced in six languages to target a large proportion of the immigrant population.
- Web-based educational materials on antibiotic use are disseminated to both clinicians and the public via websites, social media and information at health-care centres.
- Targeted information materials are distributed at local educational meetings for staff working with pre-school children or elderly people.
- Materials are created for nurses providing education about common infections to parents of newborns at child health-centres and for schoolchildren. These are created by the local group in Region Halland^41^ and available from their website as well as http://www.strama.se.
- Awareness campaigns are coordinated with the European antibiotic awareness day. In 2015, *Save the antibiotics* was launched with its own webpage (http://www.folkhalsomyndigheten.se/skyddaantibiotikan).
- A national website with information for the public about disease symptoms and health care has been created (http://www.1177.se) and1177 is also a telephone number for patients seeking advice. For triage nurses providing this advice to patients via telephone or the Internet, 1177 has built a tool to guide their advice. The website builds on a quality-controlled medical database, shared by all counties in Sweden. The 1177 website developers collaborate closely with the Swedish strategic programme against antibiotic resistance.

The following lessons were learnt about communication. First, to achieve behavioural change in the general public, it is important to communicate the negative effects for the individual of unnecessary use of antibiotics, e.g. in common upper respiratory infections. Second, to maintain public awareness about the issue, continuous communication efforts are essential, e.g. via the national and local media, about the health economic consequences and trends in antibiotic resistance. Third, for prescribers, easily accessible summaries of guidelines for common infections have been well received and used.

## Next steps

Overall use of antibiotics in Sweden over the past 20 years has decreased, without measurable negative consequences, and levels of antibiotic resistance are low compared with other countries. This can be attributed to committed work among many different professions. Antibiotic resistance cannot be eliminated, but the problem can be managed and the risks and consequences mitigated. Continuing, strong political support is therefore essential for sustainable funding for the programme. We believe that a key element for achieving long-term changes has been the bottom-up approach, working closely with prescribers at the local level. Future work will include building on this and working to achieve a sustainable approach to controlling antibiotic resistance (Box 6).

Box 6 **Future work of the strategic programme against antibiotic resistance in Sweden**

- Establish uniform long-term funding for local multiprofessional groups involved in the strategic programme.
- Maintain interest in and knowledge and understanding of antibiotic use and resistance in the next 20 years, among all stakeholders including the public. Since continued efforts depend on financial support, the issue needs to be prioritized in competition with other major health problems.
- Address barriers to implementing antibiotic stewardship programmes, involving all relevant hospital specialists, with clear objectives on how to promote the rational use of antimicrobial agents. This could include a variety of strategies, e.g. the selection of the optimal drug, dosing, duration of therapy, route of administration and the use of antibiotic susceptibility testing to reduce the use of broad-spectrum antibiotics.^10^
- Develop a national strategy for the evaluation, standardization and introduction and implementation of existing and new diagnostic methods in both inpatient and outpatient care.
- Monitor the spread of multidrug-resistant bacteria strains via travel, trade and immigration.
- Explore and evaluate the implications of increased incidence of antibiotic-resistant bacteria for infection control practices, e.g. management of the risk of spread of multidrug-resistant bacteria, due to limited numbers of single rooms and overcrowding in hospitals.
- Ensure implementation of systems for obtaining standardized data from electronic patient records on diagnosis-linked data. Analyse these in relation to quality indicators on a national and local level, including regular automatic feedback to each prescriber.
- Explore what measures and incentives are needed to be in place to safeguard access to new and older antibiotics of particular medical value, that have insufficient availability on the Swedish market.
- Increase efforts to engage new stakeholders, e.g. consumer and patient organizations, and include the issue of antibiotic resistance at all educational levels.
